# Assessing Accuracy of Genotype Imputation in American Indians

**DOI:** 10.1371/journal.pone.0102544

**Published:** 2014-07-11

**Authors:** Alka Malhotra, Sayuko Kobes, Clifton Bogardus, William C. Knowler, Leslie J. Baier, Robert L. Hanson

**Affiliations:** Phoenix Epidemiology and Clinical Research Branch, National Institute of Diabetes and Digestive and Kidney Diseases, Phoenix, Arizona, United States of America; IPO, Inst Port Oncology, Portugal

## Abstract

**Background:**

Genotype imputation is commonly used in genetic association studies to test untyped variants using information on linkage disequilibrium (LD) with typed markers. Imputing genotypes requires a suitable reference population in which the LD pattern is known, most often one selected from HapMap. However, some populations, such as American Indians, are not represented in HapMap. In the present study, we assessed accuracy of imputation using HapMap reference populations in a genome-wide association study in Pima Indians.

**Results:**

Data from six randomly selected chromosomes were used. Genotypes in the study population were masked (either 1% or 20% of SNPs available for a given chromosome). The masked genotypes were then imputed using the software Markov Chain Haplotyping Algorithm. Using four HapMap reference populations, average genotype error rates ranged from 7.86% for Mexican Americans to 22.30% for Yoruba. In contrast, use of the original Pima Indian data as a reference resulted in an average error rate of 1.73%.

**Conclusions:**

Our results suggest that the use of HapMap reference populations results in substantial inaccuracy in the imputation of genotypes in American Indians. A possible solution would be to densely genotype or sequence a reference American Indian population.

## Introduction

Current technologies allow rapid and extensive genotyping of common single nucleotide polymorphisms (SNPs) [1], [2]. However, none of the available genotyping platforms has complete coverage. Furthermore, while there has been an increase in collaborative studies (for example, genome-wide association meta-analyses), problems arise when different genotyping platforms have been used and many markers do not overlap [3]. One solution has been the development of methods to impute untyped variants using information on linkage disequilibrium (LD) with typed markers [3]–[5]. For example, using genotype imputation, a meta-analysis of type 2 diabetes genome-wide association studies was performed by Zeggini et al. and this study resulted in the identification of at least six new diabetes loci [6]. Similarly, a meta-analysis testing association of low-density lipoprotein cholesterol (LDL) to SNPs in the low-density lipoprotein receptor region on chromosome 19 showed strong evidence for association at an imputed SNP (rs5611270), which was not well tagged by any of the individual SNPs. A subsequent replication study in which this SNP was directly genotyped further confirmed association with LDL with a p<10^−25^ [4].

To impute genotypes, a suitable reference population is required, most often one selected from HapMap [7]–[9]. This reference population has all the markers of interest genotyped. The LD pattern between markers is then used to infer genotypes for untyped markers from those for the typed markers in the population being studied. While HapMap has information on a large number of ethnic groups, including African Americans and Mexican Americans, American Indians are not represented.

One study has used HapMap2 reference populations to estimate imputation accuracy in a variety of populations including a small group of American Indians from Mexico [10]. While this study concluded that using a combination of HapMap groups as the reference can provide accurate imputation in many populations (estimating allelic error rate at <5%), these results need to be validated in other studies since the sample sizes in many groups were very small. For example, accuracy in Pima Indians was estimated using only eight individuals from the Human Genome Diversity Cell Line Panel [10]. Furthermore, this study used only HapMap2 data and potentially more appropriate reference populations, such as Mexican Americans, are present in HapMap3.

In the current study, we evaluated the accuracy of imputation in 1,266 Pima Indians who had participated in a genome-wide association study (GWAS) [11]. The accuracy of imputation using HapMap reference populations was compared with that using the Pimas as their own reference. To our knowledge, this is the first extensive exploration of accuracy of HapMap populations in predicting American Indian genotypes.

## Methods

### Ethics Statement

Written informed consent was obtained for all participants and this study was approved by the institutional review board of the National Institute of Diabetes and Digestive and Kidney Diseases.

### Study population genotypes and quality control

A previous GWAS in Pima Indians (N = 1,266) provided genotype information on 454,194 SNPs [11]. The GWAS SNPs passed the following quality control criteria: Hardy-Weinberg Equilibrium p-value>0.001, SNPs were genotyped in >85% of individuals, and minor allele frequency >0.05. Due to the computational burden in imputing the entire genome, six randomly selected chromosomes were analyzed (chromosomes 1, 7, 8, 15, 17, and 22).

### Reference populations

Using HapMap2 data, we used the Yoruba (YRI; N = 120 haplotypes), White (CEU; N = 120 haplotypes), and Japanese and Chinese combination (JPT+CHB; N = 180 haplotypes) separately as reference populations [7]. In addition, we used the Mexican ancestry (MXL; N = 104 haplotypes) HapMap3 data since this group is not represented in HapMap2 [8].

We also used Pima Indians as a reference population; LD patterns in the Pima Indian GWAS were used to impute and predict genotypes. In addition, to evaluate the influence of the size of the reference Pima panel, we estimated accuracy when randomly selecting a subset of Pima individuals as the reference (N = 25, 50, 100 respectively). Haplotypic phase in the reference population was assigned using the program fastPHASE [12] prior to imputation. To assess accuracy using the Pima reference comparably to other reference populations, the phase information in the study Pima population was considered unknown.

In these analyses, individuals selected for the reference panel were excluded from assessment of genotype accuracy (ie, removed from the study population so that their genotypes do not contribute to the assessment of accuracy). Genotypes were then imputed separately using the 50, 100, or 200 haplotypes (equivalent to two haplotypes per individual).

### Imputation

All genotype imputation procedures were performed using the software MaCH (Markov Chain Haplotyping Algorithm) [13]. To evaluate accuracy, a procedure called ‘masking’ was used. In this method, randomly selected genotypes in the study population are assigned missing values, the imputation procedure is carried out, and the assigned genotypes are then compared with the original genotypes [13].

Either 1% or 20% of genotypes were masked for each chromosome. The method of imputation implemented in MaCH has been previously described [13]. Briefly, a random pair of haplotypes, that are consistent with observed (ie, 80% or 99% unmasked) genotypes, is assigned to each individual in the study population in each iteration. A previously assigned number of iterations (50 in the current study) are carried out, with the results of each iteration being stored. Once the process is complete, the most likely genotype is identified.

### Imputation quality assessment

Once genotypes were assigned to the masked genotypes, these were compared to the original genotypes. Two measures of accuracy were used as implemented in MaCH [13]:

1. The proportion of genotypes that were correctly imputed when the most likely genotype was taken, which was calculated as follows: assuming a SNP has alleles A and B, and I is the total number of iterations (i.e., (I) = n_A/A_+n_A/B_+n_B/B_. where n_A/A_, n_A/B_, n_B/B_ are the number of iterations where each genotype is assigned), the most likely genotype is taken (imputed genotype IG) as the one most commonly observed over all iterations (e.g., AA, AB or BB). From this, the proportion (P) where IG = true genotype is estimated. The genotype error rate = 1 - P.
2. The average estimated r^2^ between the true and imputed genotype is estimated by comparing the variance of genotype scores between imputed genotypes and actual genotypes. This is estimated as follows: looking at the number of copies of allele A, the genotype score (g) = (2n_A/A_+n_A/B_)/I. The variance of g (Var(g)) is then estimated and used in estimating r^2^ of variances (ie, between Var(g) and expected variance if genotype scores were observed without error).

The masking procedure implemented in MaCH masks individual genotypes and not entire SNPs. While one might expect accuracy estimates to be comparable regardless of which approach is used, in practice imputation is often performed for SNPs for which no one in the study population is typed, for example, when combining populations in a meta-analysis. To assess accuracy in the context for which a SNP is not typed in the study population, we assigned 1% of randomly selected SNPs as missing in the study population, followed by imputation from the reference population. We estimated error rates for the SNPs marked as missing by comparing the most likely imputed genotype with the observed genotype and by calculating the correlation between the expected genotype, assessed by imputation, and its observed value. In other words, the correlation between the “expected” genotype score (g = (2n_A/A_+n_A/B_)/I) and the observed number of A alleles (coded 0, 1, 2) was calculated. Since the error rates calculated by this method were comparable to the error rates observed when individual genotypes were masked, and since masking individual genotypes may provide a wider representation of the sample that is not subject to individual SNP differences, we only report the results for masking individual genotypes.

## Results and Discussion

**Figure 1:**
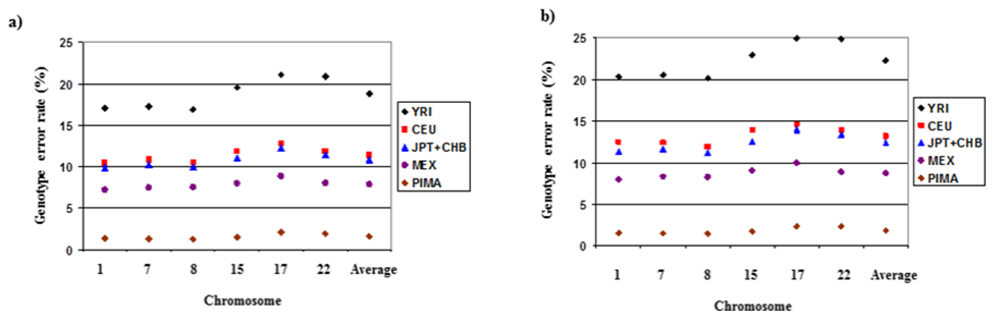
Genotype error rates according to reference population when a) 1% or b) 20% of genotypes were masked.

Figure 1 shows the results for the analyses with 1% and 20% of SNPs masked, respectively. Using the HapMap reference populations, the MXL population gave the lowest genotype error rate, followed by JPT+CHB and CEU. The YRI reference population gave the highest error rate. Using the MXL population, when 1% of SNPs were masked, the average genotype error rates ranged from 7.24% to 8.85% depending on the chromosome being analyzed. As might be expected, the error rates were slightly higher when 20% of markers were missing with results ranging from 7.97% to 9.97% in MXL, 11.22% to 13.98% in JPT+CHB, 11.74% to 13.65% in CEU, and 19.22% to 24.93% in YRI.

**Figure 2:**
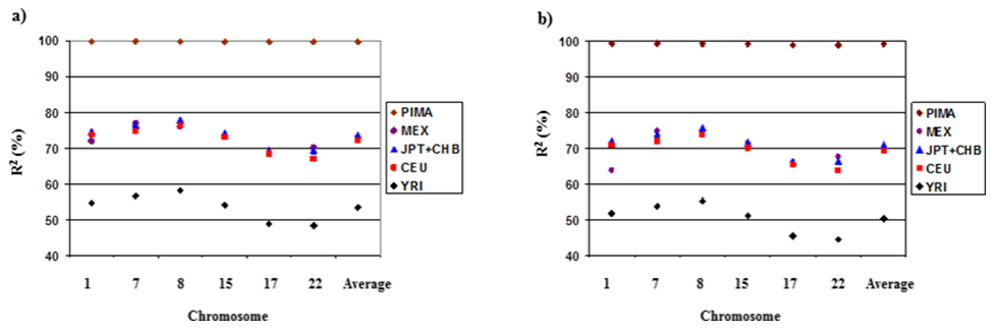
R^2^ according to reference population when a) 1% or b) 20% of genotypes were masked.

Figure 2 shows the r^2^ results by reference population and chromosome. Using the MXL, JPT+CHB, and CEU reference populations gave similar r^2^ values with averages of 0.73, 0.74, and 0.73 in the 1% masking analyses, respectively. The YRI population showed much lower r^2^ values ranging from 0.49 to 0.58. A value of <0.30 is often used as criteria to reject use of a SNP. For the 1% masking analyses, an average of 17.96%, 15.89%, 16.26%, 35.96% of SNPs had r^2^<0.3 in MXL, JPT+CHB, CEU, and YRI, respectively.

The results described above show that from the HapMap populations, the MXL reference provides the most accurate imputation results for Pima Indians compared to CEU, YRI, or JPT+CHB. HapMap3 contains fewer markers than HapMap2 and it is possible that these panels may perform differently in assigning haplotypes. To address the possibility of specific differences between HapMap2 and HapMap3 being responsible for the higher accuracy in MXL, we tested the CEU and JPT+CHB HapMap3 data as well. The results were similar to those obtained for HapMap2 (results not shown).

Figures 1 and 2 also show the genotype error rates and r^2^ when using Pima Indians as a reference population (100 randomly selected individuals from the GWAS study). Using Pima Indian data significantly reduces the error rates and improves r^2^. An average genotype error rate of 1.59% and 1.87% was observed when 1% and 20% of SNPs were masked, respectively. Furthermore, r^2^ of >0.98 was estimated in all cases.

Combining multiple HapMap populations has been proposed in a previous study as the optimal approach for many populations, including American Indians [10]. We tested this by using two sets of populations: 1) combining CEU, YRI, and JPT+CHB from HapMap2 and 2) combining CEU, YRI, JPT+CHB, and MXL from HapMap3. Due to computational burden, we limited our analyses to three chromosomes. The genotype error rates in all cases were higher than those observed using the MXL population alone or the Pima-specific reference, with average error rates of 11.37% and 9.76% for the combined HapMap2 and HapMap3 populations, respectively. Therefore, from all HapMap populations tested in the current study, use of data from the Mexican American population alone would provide the most appropriate reference for genotype imputation in Pima Indian populations. Furthermore, the error rates observed in the present study in all cases were higher than those observed in Pima Indians by Huang et al in analyses that included data from eight Pima Indian individuals [10]. Their small sample size might explain the differing results with the current study. It is also possible that maximizing the predictive properties for a given sample over multiple reference populations can result in an overestimate of the accuracy observed in a new sample.

**Figure 3:**
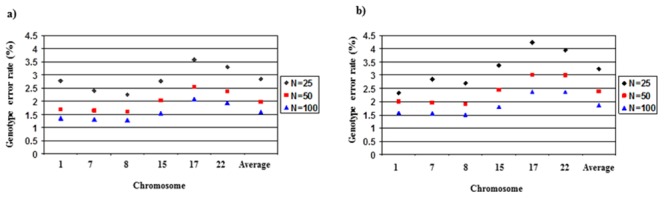
Genotype error rates according to Pima Indian reference population size when a) 1% or b) 20% of genotypes were masked.

**Figure 4:**
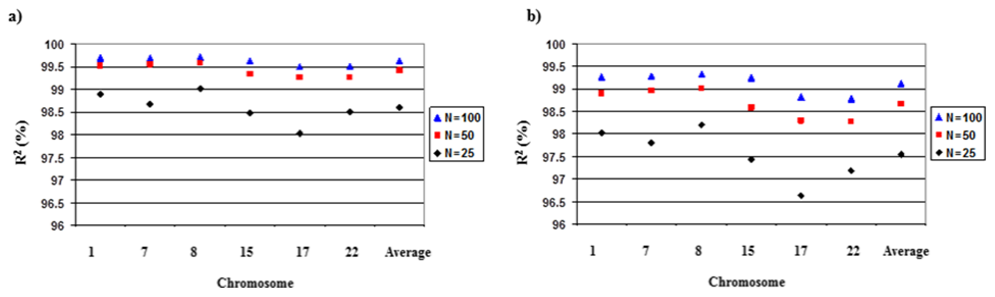
R^2^ according to Pima Indian reference population size when a) 1% or b) 20% of genotypes were masked.

In the present study, using Pima Indians as a reference population significantly improves imputation accuracy. Therefore, the ideal situation would be to have a population specific reference group to accurately impute untyped genotypes. We further explored the extent to which the number of individuals used in the Pima reference population influenced accuracy. As we described earlier, there were 120, 180, 120, and 104 haplotypes in the CEU, JPT+CHB, YRI, and MXL reference populations. To perform comparable analyses, we performed imputation analyses using three reference groups (with 25, 50, or 100 individuals) of randomly selected Pima Indians from the original GWAS study (N = 1266). Using these subsets of the Pima dataset, average genotype error rates were 2.84%, 1.98%, 1.59%, respectively for 25, 50, and 100 reference Pima individuals in the 1% masking analyses, with r^2^>0.97 in all cases. R^2^ values were similar in the 20% masking analyses, with slightly higher genotyping error rates (Figures 3 and 4). These results show that imputation with a reference population of only 25 individuals was considerably more accurate than with any of the HapMap reference populations.

The Pima Indians for the GWAS were selected from sibships, and the presence of closely related family members may result in increased imputation accuracy. To evaluate this, we also assessed the accuracy of imputation after removing first-degree relatives of individuals in the reference population from the study population. The results were similar whether or not these relatives were included (results not shown).

To assess the potential implications of the present findings on statistical power, we performed a power analysis using the genetic power calculator [14] assuming singleton data from 1266 individuals. For a type 1 error rate of 10^−3^, we estimated the power to detect an association with a quantitative trait, assuming that the SNP explains 1% of the variance. Average power for an imputed SNP was estimated at 41% for MXL, CEU, and JPT+CHB references, 25% for a YRI reference, 60% using a Pima reference, and 61% assuming the SNP is directly genotyped (ie, r^2^ = 1). To achieve 80% power, the variance explained by the imputed SNP would have to be approximately 1.8% for MXL, CEU, and JPT+CHB references, 2.5% for a YRI reference, and 1.35% using a Pima reference. This compares with 1.33% assuming the SNP is directly genotyped.

In the absence of a closely related reference population that is densely typed for many markers, investigators will often choose the most closely-related HapMap population, or a combination of HapMap populations, as a reference for imputation. However, the magnitude of imputation inaccuracy observed in the present study suggests that this approach will result in a significant loss of power, and, thus, it may be worthwhile to develop a population-specific reference panel. Power of genome-wide association studies using imputed genotypes has been assessed in previous studies [15], [16]. In a study looking at the relationship between imputation error rate and sample size requirements, Huang et al showed that error rates of 2–6% allelic error rate can result in up to 40–60% increase in sample size to achieve the same power if the SNPs were directly genotyped [16]. Their estimate of the sample size inflation rate in Mexican Pima Indians was ∼1.25 based on eight individuals from the Human Genome Diversity Cell Line Panel. However, they estimated allelic error rates of ∼3%, which are lower than seen in the current study. Therefore, it seems reasonable to assume that given the high error rates observed in the current study for a majority of HapMap reference populations, the sample size required to achieve good power would greatly increase.

We recognize that the current imputation study has some limitations. The use of genotype data from this genome-wide association study might not be ideal as a reference population since the individuals were not randomly selected, but were chosen based on a clinical phenotype. It might be more appropriate to include an independent Pima Indian population genotyped for the same SNPs available in HapMap2 or HapMap3 [8], [9].

Furthermore, the data were obtained from the Affymetrix array, and it is possible that other SNPs would have different characteristics. A potential concern is that markers present on the Affymetrix array, but not included in HapMap2 and HapMap3, are only available in the Pima reference sample, and these additional markers may account in part for the improved accuracy for the Pima reference compared with the other reference populations. To address this possibility, we re-analyzed the data and performed imputation using only those SNPs that overlapped in all HapMap populations and the Pima Indian population. These analyses showed the same order of accuracy (ie, Pima reference populations gave the lowest error rate followed by Mexican Americans, with the other HapMap populations showing higher error rates). Furthermore, in all cases, the genotype accuracy rates were on average only slightly decreased due to the use of a smaller SNP set. Finally, with our present data we cannot determine the accuracy of any of the reference populations, including MXL and Pimas, for imputation in other American Indian populations.

While genotype imputation has resulted in increased information in association studies, there is a need for caution in some cases. This was illustrated in a paper by Beecham et al describing joint analysis of multiple studies using genotypes from different platforms [17]. The joint analysis showed no evidence for association between late-onset Alzheimer disease and the *apolipoprotein-e* (*APOE*) gene, a locus that has been replicated in numerous studies [18]. The reason for this discrepancy was due to the weak linkage disequilibrium of SNPs near the *APOE* locus. The authors proposed that genotype uncertainty should be accounted for in the meta-analysis [17].

In addition to imputation of common variants, which are generally used in genome-wide association studies, there is increasing interest in imputation of rare variants from sequencing data. Fridley et al. have explored cost-effective ways to impute rare variants and suggest using sequence data from the 1000 genomes project and possibly combining this information with actual sequence data from a subset of the population being studied [19]. This approach might work in Pima Indians. However, given that high error rates observed with HapMap reference populations, it is likely that error rates using data from the 1000 genomes project will also be high given the lack of representation of American Indians. Therefore, it would be preferable to sequence a small number of Pima Indians as a reference population, which will allow imputation of both common and rare variants in this population.

In conclusion, using Pima Indians as a reference population significantly improves imputation accuracy when compared to HapMap populations. There is a need to also develop a American Indian reference panels to accurately impute untyped genotypes in these populations.

### Data Availability Statement

The IRB has determined that the consent does not allow for placement of individual-level data in a public repository. However, these data may be available to researchers if a data use agreement is signed (which is subject to IRB approval). Interested investigators should contact the authors.

## References

[1] Ding C, Jin S (2009) High-throughput methods for SNP genotyping. Methods Mol Biol 578: 245–254. 19768599 10.1007/978-1-60327-411-1_16

[2] Ragoussis J (2009) Genotyping technologies for genetic research. Annu Rev Genomics Hum Genet 10: 117–133. 19453250 10.1146/annurev-genom-082908-150116

[3] Marchini J, Howie B (2010) Genotype imputation for genome-wide association studies. Nat Rev Genet 11: 499–511. 20517342 10.1038/nrg2796

[4] Li Y, Willer C, Sanna S, Abecasis G (2009) Genotype imputation. Annu Rev Genomics Hum Genet 10: 387–406. 19715440 10.1146/annurev.genom.9.081307.164242 PMC2925172

[5] Pei YF, Zhang L, Li J, Deng HW (2010) Analyses and comparison of imputation-based association methods. PLoS One 5: e10827. 20520814 10.1371/journal.pone.0010827 PMC2877082

[6] Zeggini E, Scott LJ, Saxena R, Voight BF, Marchini JL, et al (2008) Meta-analysis of genome-wide association data and large-scale replication identifies additional susceptibility loci for type 2 diabetes. Nat Genet 2008 40: 638–645. 10.1038/ng.120 PMC2672416 18372903

[7] The International HapMap Consortium (2003) The International HapMap Project. Nature 426: 789–796. 14685227 10.1038/nature02168

[8] The International HapMap Consortium (2007) A second generation human haplotype map of over 31 million SNPs. Nature 449: 851–861. 17943122 10.1038/nature06258 PMC2689609

[9] The International HapMap 3 Consortium (2010) Integrating common and rare genetic variation in diverse human populations. Nature 467: 52–58. 20811451 10.1038/nature09298 PMC3173859

[10] Huang L, Li Y, Singleton AB, Hardy JA, Abecasis G, et al (2009) Genotype-imputation accuracy across worldwide human populations. Am J Hum Genet 84: 235–250. 19215730 10.1016/j.ajhg.2009.01.013 PMC2668016

[11] Malhotra A, Kobes S, Knowler WC, Baier LJ, Bogardus C, et al (2011) A genome-wide association study of BMI in American Indians. Obesity 19: 2102–6. 21701565 10.1038/oby.2011.178 PMC7229868

[12] Scheet P, Stephens M (2006) A fast and flexible statistical model for large-scale population genotype data: applications to inferring missing genotypes and haplotypic phase. Am J Hum Genet 78: 629–644. 16532393 10.1086/502802 PMC1424677

[13] Li Y, Willer CJ, Ding J, Scheet P, Abecasis GR (2011) MaCH: using sequence and genotype data to estimate haplotypes and unobserved genotypes. Genet Epidemiol 34: 816–834. 10.1002/gepi.20533 PMC3175618 21058334

[14] Purcell S, Cherny SS, Sham PC (2003) Genetic Power Calculator: design of linkage and association genetic mapping studies of complex traits. Bioinformatics 19: 149–150. 12499305 10.1093/bioinformatics/19.1.149

[15] Anderson CA, Pettersson FH, Barrett JC, Zhuang JJ, Ragoussis J, et al (2008) Evaluating the effects of imputation on the power, coverage, and cost efficiency of genome-wide SNP platforms. Am J Hum Genet 83: 112–119. 18589396 10.1016/j.ajhg.2008.06.008 PMC2443836

[16] Huang L, Wang C, Rosenberg NA (2009) The relationship between imputation error and statistical power in genetic association studies in diverse populations. Am J Hum Genet 85: 692–698. 19853241 10.1016/j.ajhg.2009.09.017 PMC2775841

[17] Beecham GW, Martin ER, Gilbert JR, Haines JL, Pericak-Vance MA (2010) APOE is not associated with Alzheimer disease: a cautionary tale of genotype imputation. Ann Hum Genet 74: 189–194. 20529013 10.1111/j.1469-1809.2010.00573.x PMC2934779

[18] Bertram L, McQueen MB, Mullin K, Blacker D, Tanzi RE (2007) Systematic meta-analyses of Alzheimer disease genetic association studies: the AlzGene database. Nat Genet 39: 17–23. 17192785 10.1038/ng1934

[19] Fridley BL, Jenkins G, Deyo-Svendsen ME, Hebbring S, Freimuth R (2010) Utilizing genotype imputation for the augmentation of sequence data. PLOS ONE 5: e11018. 20543988 10.1371/journal.pone.0011018 PMC2882389

